# Artificial Hybrids of Influenza A Virus RNA Polymerase Reveal PA Subunit Modulates Its Thermal Sensitivity

**DOI:** 10.1371/journal.pone.0015140

**Published:** 2010-12-07

**Authors:** Takahito Kashiwagi, Koyu Hara, Yoko Nakazono, Nobuyuki Hamada, Hiroshi Watanabe

**Affiliations:** Division of Infectious Disease, Department of Infectious Medicine, Kurume University School of Medicine, Kurume, Japan; University of Cambridge, United Kingdom

## Abstract

**Background:**

Influenza A virus can infect a variety of different hosts and therefore has to adapt to different host temperatures for its efficient viral replication. Influenza virus codes for an RNA polymerase of 3 subunits: PB1, PB2 and PA. It is well known that the PB2 subunit is involved in temperature sensitivity, such as cold adaptation. On the other hand the role of the PA subunit in thermal sensitivity is still poorly understood.

**Methodology/Principal Findings:**

To test which polymerase subunit(s) were involved in thermal stress we reconstituted artificial hybrids of influenza RNA polymerase in ribonucleoprotein (RNP) complexes and measured steady-state levels of mRNA, cRNA and vRNA at different temperatures. The PA subunit was involved in modulating RNP activity under thermal stress. Residue 114 of the PA subunit was an important determinant of this activity.

**Conclusions/Significance:**

These findings suggested that influenza A virus may acquire an RNA polymerase adapted to different body temperatures of the host by reassortment of the RNA polymerase genes.

## Introduction

In April 2009, the Centers for Disease Control and Prevention (CDC) reported that newly influenza A virus (H1N1) has emerged in Mexico [1]. Influenza A virus had quickly spread worldwide [2] and the World Health Organization (WHO) declared a pandemic phase 6 [3]. Currently, this new pandemic influenza A virus is still circulating around the world replacing the seasonal "Russian" influenza A (H1N1) [3]. The H1N1 pandemic virus was immediately characterized [4] and determined as a triple reassortant derived from human, avian and swine influenza viruses [5], [6]. WHO announced that the virus was a low pathogenic virus based on the amino acid features of both HA and PB2 genes, and the fatality rate of this virus was also low [3]. Although phylogenetic relationships of the 8 gene segments were quickly and easily obtained [5], [7], the primary host is still uncertain even though the virus quickly established infection in swine [8], [9]. Thus it will be difficult to predict the exact origin of the next pandemic influenza strain as previous predictions of a pandemic avian-derived H5N1 virus proved unfounded [10].

One of the approaches to solve the problem of predicting new pandemics is to study host restriction, because the route of the infection from animals to humans is an important factor in the emergence of a new pandemic virus. Although some determinants of host restriction, such as the type of sialic acid on the cell surface [11], the ease of dissociation of HA by host proteases [11], the interaction between PB2 and alpha-importins [12], [13] and the amino acid position 627 on PB2 [14], [15] are well known, we have focused on the relationship between the host's body temperature and the viral RNA polymerase, because the influenza virus is a disease affecting animals and birds with different host temperatures. A study of the RNA polymerase, comprised of 3 different subunits, is particularly relevant to swine flu since this newly emerged H1N1 swine virus is a hybrid RNA polymerase derived from humans and birds [5] whose body temperatures differ. Here we test the hypothesis that the optimal temperature of influenza A RNA polymerase can be modulated by the differing combination of its polymerase subunits resulting in a virus adapted to different body temperatures.

The influenza A virus RNA polymerase is a trimeric complex of three different subunits - PB1, PB2 and PA, which in association with the nucleoprotein and viral RNA form the active ribonucleoprotein complex (RNP) [11], [16]. Recently, high resolution structures of amino acids 1-197 of the N-terminal region of PA subunit [17], amino acids 257-716 of PA, complexed with a short peptide at the N-terminus of PB1 [18] and short regions of the structure of PB2 [19], [20], [21] have been determined. All three subunits are generally found to be required for both transcription and replication [11], [16], although other reports disagree [22]. In addition, the influenza RNA polymerase is required not only for the RNA replication, but also for the thermal sensitivity. In fact, the avian PB2 subunit is required for replication at high temperatures [23], presumably because of the high body temperature of birds. Moreover, the PB2 subunit is associated with the efficient replication of cold adapted influenza virus [24], [25], [26]. In influenza B virus, the PB2 gene was also involved in cold-adaptation [27]. Thus the PB2 subunit has a crucial role for the thermal activity of the RNA polymerase.

Additional functions of the PA subunit of the RNA polymerase have recently been identified including its role in transcription [11], [16], replication [11], [16], endonuclease activity [28], [29], [30], cap binding [30], promoter binding [30], [31], [32], [33], proteolytic activity [34], [35] and virulence for mice [36]. Thus the PA subunit has a crucial role in RNA replication and viral proliferation, but its function in host restriction and thermal stability are still poorly understood. Recently, it has been shown that the steady-state level of polymerase - cRNA complex is important for the thermal stability of the replication [37]. On the other hand we have previously reported that the PA subunit of the influenza RNA polymerase is required for promoter binding to cRNA [31], [32]. Taken together, we speculated that there is the relationship between thermal stability of cRNA and the PA subunit of the RNA polymerase.

Accordingly, to investigate whether the PA subunit is an important factor for replication and transcription under thermal stress, we focused on the PA subunit and measured the replication and transcription activity of RNP in 293T cells at various temperatures (34°C - 42°C). Our results suggested the PA was involved in modulating RNP activity at different temperatures, and that position 114 of PA was one of the important determinants.

## Materials and Methods

### Strains

RNA or cDNA clones isolated from the following influenza strains were used: A/HongKong/156/97 (H5N1) (abbreviated as HK), A/Vietnam/1194/04 (H5N1) (abbreviated as VN), A/WSN/33 (H1N1) (abbreviated as WSN), A/NT/60/68 (H3N2) (abbreviated as NT). Newly pandemic influenza A virus (H1N1) was isolated from outpatient in Kurume university hospital and named as A/Kurume/K0910/2009 (H1N1) (abbreviated as SW). Position 627 of the PB2 subunit in HK and SW is glutamic acid (E); the PB2 subunits of the other strains have a lysine (K) at position 627.

### Plasmids

PB1, PB2, PA and NP-expressing plasmids of influenza viruses WSN, HK, VN and NT have previously been described [31], [38] and the pPOLI-vNA plasmid has also been described previously [31], [39]. To construct point mutants of each PA subunit, mutagenesis method was used as previously reported [30], [31]. WSN NP was used in all experiments, according to previous report [31].

To construct PB1, PB2 and PA expression vectors of SW, RT-PCR was performed with Superscript II reverse transcriptase (Invitrogen) and PFU Turbo (Stratagene) with RNA isolated from outpatient in Kurume University. PCR fragments were digested by EcoRV and XhoI and inserted into pcDNA3A(-) (Invitrogen), generating pcDNA/SW/PB1, pcDNA/SW/PA, pcDNA/SW/PB2 and pcDNA/SW/PB2-TAP. The isolated SW PB1 [accession number HM849035] contains 3 mutations of G1299A, A1303G and A1758G (counting from the A of the initiator ATG as nucleotide 1) compared to the sequence of A/California/04/2009 PB1 [accession number FJ966080] [5], [8], causing 1 coding change of I435V. The SW PA [accession number HM849037] also has 2 mutations of C670T and T1986G compared to the sequence of A/California/04/2009 PA [accession number FJ966081] [5], [8], generating 1 coding change of P224S. In addition, the SW PB2 [accession number HM849036] possesses 4 mutations of G752A, C1873T, G2010A and G2164A compared with A/California/04/2009 PB2 [accession number FJ966079] [5], [8], generating 1 coding change of R251K.

### Preparation of partially purified TAP-tagged polymerase and *in vitro* assays

293T cells were transfected with the expression vectors containing PB1, PB2-TAP and PA subunit of each strain on 10 cm dishes [30], [31], [38]. Crude cell lysates were harvested at 40 hours post-transfection and the polymerase were partially purified by the tandem affinity purification (TAP) method described previously [30], [31], [38]. The partially purified polymerase was analyzed by 7.5% SDS-PAGE with silver staining (Invitrogen) and confirmed by western blotting with specific antibody against PB1, PB2 and PA [34], [40].

The dinucleotide initiation of replication assay was performed as described previously [41], [42], and UV cross-linking to model vRNA and cRNA promoters was also performed as reported previously [29], [31], [32].

### RNA isolation and primer extension assay in 293T cell

Subconfluent monolayer of 293T cells in E-MEM medium supplemented with 10% fetal bovine serum, in 6 wells plate were transfected with Lipofectamine 2000 reagent (Invitrogen) according to the manufacture's protocol [30], [31], [39]. 0.2 µg each of PA, PB1, PB2, NP and vNA (viral NA gene) expression vector of each strain (WSN, NT, HK, VN or SW) were diluted with 50 µl OPTI-MEM (Invitrogen). This solution was then mixed with 4 µl of Lipofectamine 2000 reagent (Invitrogen) previously diluted in 250 µl OPTI-MEM. For the thermal stress, the cells were incubated at 37°C as pre-incubation for 24 hours and then transferred to 34, 37 or 42°C for several hours based on each experiment. Later, total cell RNA was extracted using TRIzol reagent (Invitrogen). RNA was then analyzed in a primer extension assay using three primers-one for vRNA, one for mRNA and cRNA, one for 5S rRNA as an internal control [29], [31], [32]. Transcripts were visualized by 6% polyacrylamide gel containing 7 M urea in TBE buffer and quantitated by autoradiography and Quantity One software version 4.6.7 (Bio-Rad). The total lysate including influenza RNP was analyzed by 7.5% SDS-PAGE with silver staining (Invitrogen) and confirmed by western blotting with specific antibody against PB1, PB2, PA and NP [34], [40].

## Results

### Comparison of RNP activity of different viruses under thermal stress

**Figure 1:**
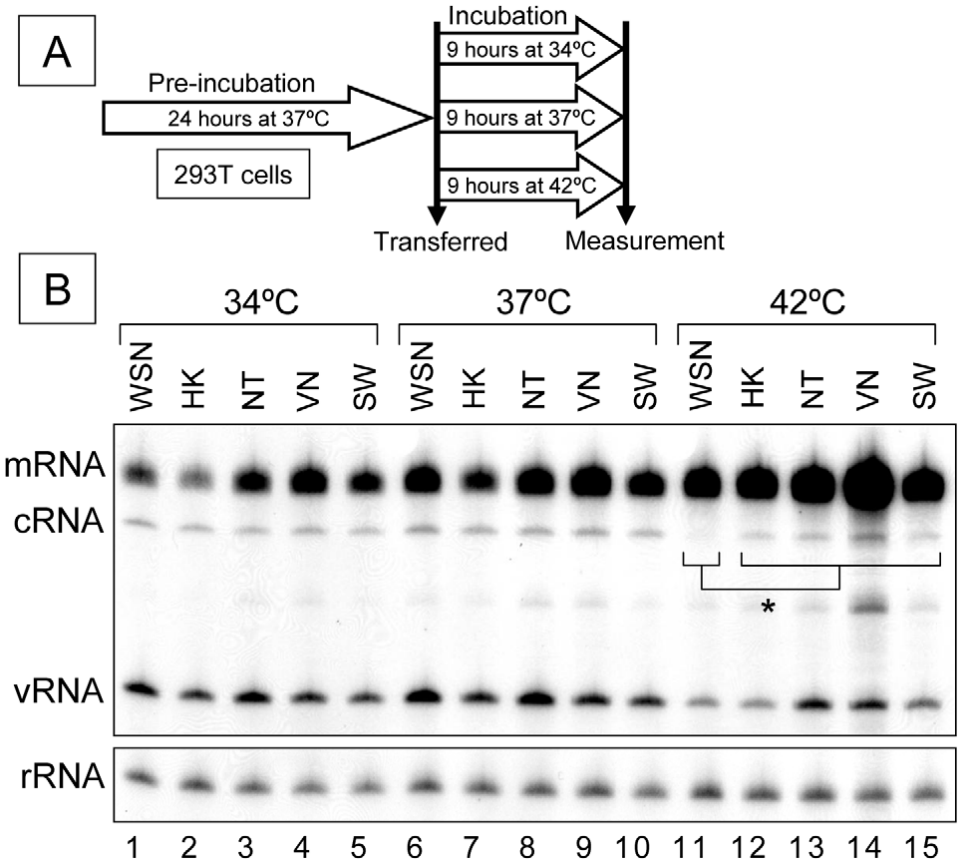
Comparison of RNP activities under thermal stress. (A) Brief protocol and incubation periods are indicated. (B) Representative analyzed polyacrylamide gel (6%) is shown. * represents statistical significance at p<0.05 in a Student's t-test (n = 3). 293T cells expressing influenza RNP were incubated at 37°C for 24 hours as pre-incubation. Pre-incubated cells were additionally incubated at 34, 37 and 42°C for 9 hours, respectively. Then total RNAs were extracted and analyzed by primer extension assay. A/WSN/33, A/Hong Kong/156/97, A/NT/60/68, A/Vietnam/1194/2004 and pandemic H1N1 2009 virus are abbreviated as WSN, HK, NT, VN and SW, respectively. 5s ribosomal RNA (rRNA) is indicated as an internal control. mRNA, cRNA and vRNA are viral messenger RNA, complementary viral RNA and viral RNA, respectively.

To test if thermal stress affects RNA polymerase activity, 293T cells expressing influenza A ribonucleoprotein (RNP) of 5 different viruses isolated from humans were incubated at various temperatures from 34°C to 42°C for 9 hours, following pre-incubation at 37°C for 24 hours (Figure 1A). A/Hong Kong/156/97 (H5N1) [HK] and A/Vietnam/1194/2004 (H5N1) [VN] were chosen as human-isolated H5N1 viruses; A/WSN/33 (H1N1) [WSN] and A/NT/60/68 (H3N2) [NT] were used as classical human strains, because these strains were previously extensively analyzed [31], [32], [39]. We included the new pandemic influenza A virus, A/Kurume/K0910/2009 (H1N1) [SW], cloned from an outpatient in Kurume university hospital, Japan. We analyzed steady-state levels of mRNA, cRNA and vRNA by primer extension (Figure 1B) as described before (see materials and methods) and summarized in Table 1. All strains were pleiotropic in RNP activity when exposed to thermal stress from 34°C to 42°C (Figure 1B and Table 1).

**Table 1:** Relative RNP activities of each strain at different temperatures.

	mRNA					cRNA					vRNA				
(%^a^)/°C	34		37	42		34		37	42		34		37	42	
WSN	52	±7.8	100	129	±13.0	76	±12.6	100	31	±18.3	86	±8.6	100	38	±5.5
HK	54	±5.6	100	221	±38.9	84	±5.0	100	78	±18.3	89	±4.5	100	54	±4.9
NT	85	±18.8	100	163	±13.4	100	±35.3	100	125	±40.9	90	±8.3	100	79	±7.7
VN	82	±1.1	100	170	±9.8	90	±4.2	100	224	±22.0	88	±6.2	100	86	±20.0
SW	77	±8.5	100	150	±8.2	87	±11.8	100	102	±30.8	86	±13.9	100	70	±8.2

a The replication (cRNA and vRNA) and transcription (mRNA) activities are indicated as a relative activity (%) ± S.D. to each strain set at 100% at 37°C (n = 3).

Initially, we compared the RNP activity of each strain at the classical temperature, 37°C (Figure 1B, lanes 6-10). On replication (cRNA and vRNA synthesis) at 37°C, the HK strain showed the lowest activity (Figure 1B, lane 7). Interestingly, the VN strain showed higher activity in both replication (cRNA and vRNA) and transcription (mRNA) than the HK strain, although both strains were human-isolated H5N1 virus (Figure 1B, lanes 7 and 9). However, the steady-state levels of mRNA, cRNA and vRNA of the WSN strain were significantly higher than those of the HK strain (compare Figure 1B, lanes 6 and 7), consistent with our previous report [31]. At 34°C, all strains had lower activity, although the relative activities of the 5 strains were similar at 34°C and 37°C, (Figure 1B, lane 1-5). At 42°C (Figure 1B, lanes 11-15) surprisingly, WSN cRNA synthesis was minimal (Figure 1B, lane 11), whereas vRNA synthesis was still maintained. Transcriptions (mRNA levels) were significantly enhanced at higher temperatures (42°C) in all strains (Figure 1B, lanes 11-15). In summary (Table 1) replication (especially in cRNA) of the VN and NT strains was adapted to 42°C whereas the WSN and HK strains were not well adapted to this higher temperature; the SW strain possessed an intermediate activity under thermal stress.

### Time course of RNP activity on thermal stress at 42°C

**Figure 2:**
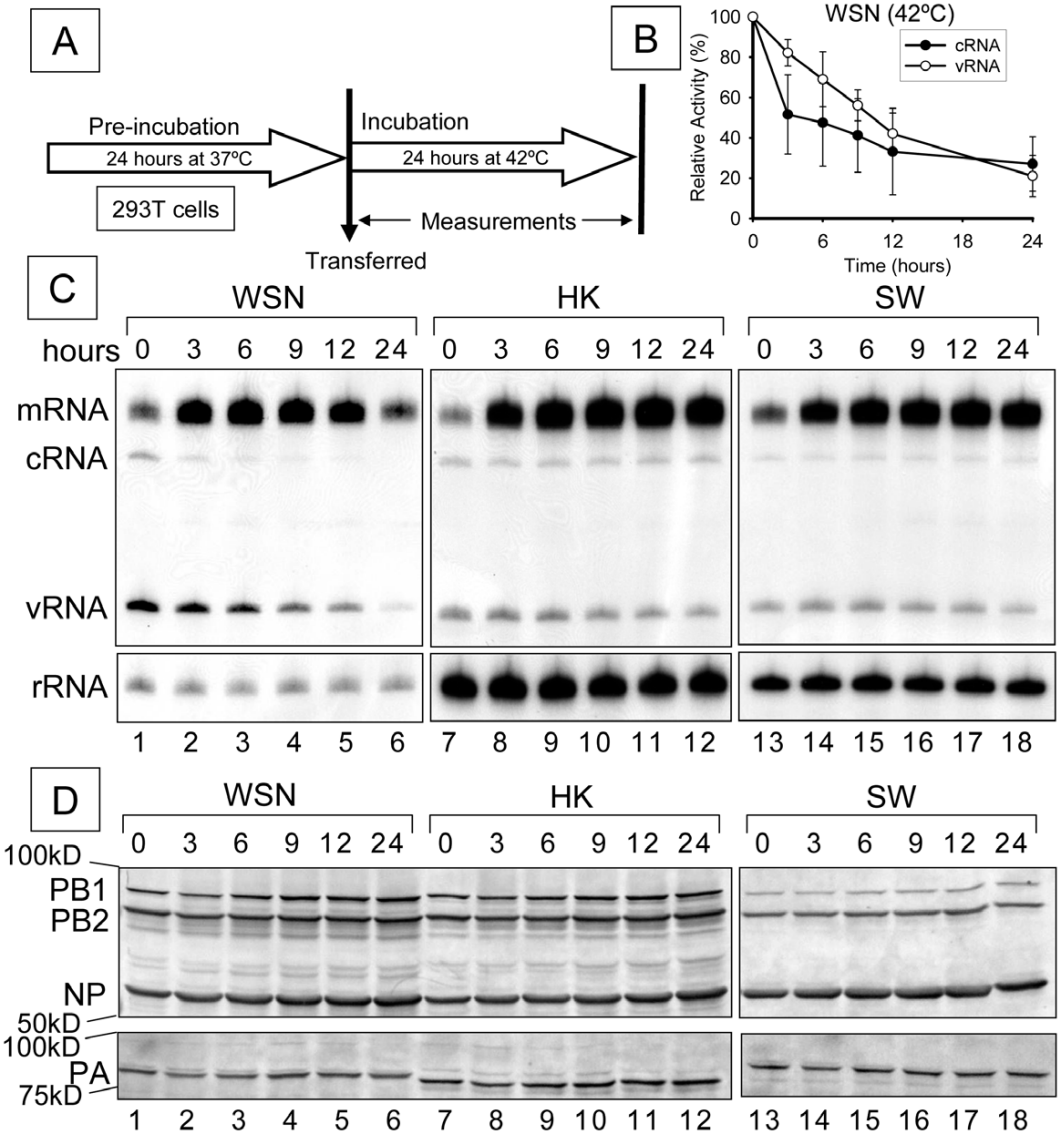
Time course of RNP activity on thermal stress at 42°C. (A) Brief protocol and incubation periods are indicated. (B) Quantitation of cRNA (closed circle) and vRNA (opened circle) of WSN at 42°C is shown. Data have been normalized for total RNA using the 5S rRNA signal. Data are expressed as a percentage of wild type activity (with standard deviation). Quantitation was based on at lease three independent sets of data. (C) Representative analyzed polyacrylamide gel (6%) is shown (n = 3). 293T cells expressing influenza RNP were incubated at 37°C for 24 hours as pre-incubation. Then pre-incubated cells were transferred to 42°C. Total RNAs were extracted and analyzed at each time point by primer extension assay. (D) RNA polymerase subunits (PA, PB1 and PB2) and NP - transiently expressed in human 293T cells, analyzed by western blotting using specific anti-bodies of each subunit by 8% SDS-PAGE. A/WSN/33, A/HongKong/156/97 and pandemic H1N1 2009 virus are abbreviated as WSN, HK and SW, respectively. 5s ribosomal RNA (rRNA) is indicated as an internal control. mRNA, cRNA and vRNA are viral messenger RNA, complementary viral RNA and viral RNA, respectively.

To test whether the decreased cRNA and increased mRNA levels observed at high temperature (42°C) were time-dependent, 293T cells, transfected with expression plasmids for each RNP of WSN, HK or SW, were transferred to 42°C, after pre-incubation at 37°C for 24 hours. The mRNA, cRNA and vRNA levels were measured from 0 to 24 hours (Figure 2A) and quantitated in Figure 2B. cRNA levels of WSN rapidly decreased under thermal stress, although vRNA levels decreased more slowly (Figure 2B and Figure 2C, lanes 1-6). By contrast, the replication of both HK and SW (cRNA and vRNA) remained relatively constant over the 24 hour period (Figure 2C, lanes 7-12 and 13-18). In WSN, mRNA levels peaked at 9 hours and then steadily decreased (Figure 2C, lanes 1-6); however, mRNA levels were maintained in both HK and SW (Figure 2C, lanes 7-12 and 13-18). Expression levels of RNA polymerase subunits (PA, PB1 and PB2) and NP were shown by western-blotting in Figure 2D. Each protein tested here was maintained or slightly accumulated.

### Effect of transient heat shock at 42°C on RNP activity

**Figure 3:**
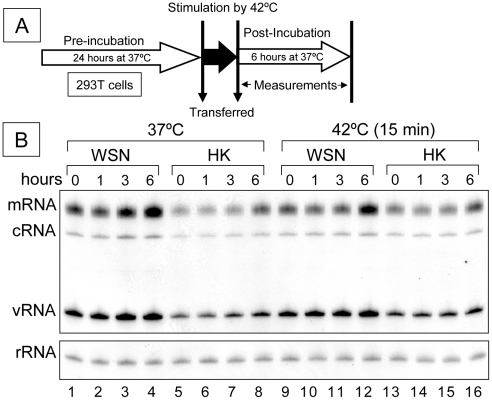
Effect of transient heat shock at 42°C on RNP activity. (A) Brief protocol and incubation periods are indicated. (B) Representative analyzed polyacrylamide gel (6%) is shown (n = 3). 293T cells expressing influenza RNP were incubated at 37°C for 24 hours as pre-incubation. Pre-incubated cells were stimulated by 42°C for 15 min, and then continued to incubate at 37°C up to 6 hours. Total RNAs were extracted and analyzed by primer extension assay. A/WSN/33 and A/HongKong/156/97 are abbreviated as WSN and HK, respectively. 5s ribosomal RNA (rRNA) is indicated as an internal control. mRNA, cRNA and vRNA are viral messenger RNA, complementary viral RNA and viral RNA, respectively.

Heat shock proteins such as Hsp70 and Hsp90 are known to be involved in viral replication [43], [44], RNA polymerase assembly [45], RNA import [45] and RNA export [46]. To investigate whether heat shock is involved directly in increasing the levels of mRNA and decreasing levels of cRNA seen above (Figures 1 and 2), 293T cells - expressing influenza RNP of WSN or HK, were heat-shocked transiently for 15 min at 42°C (Figure 3A). Both replication (cRNA and vRNA) and transcription (mRNA) were slightly elevated by 6 hours in both heat shocked and control, non heat-shocked cells that were kept at 37°C (Figure 3B). However, no significant differences were observed on quantitation of the Results (Table 2). Thus a short heat shock at 42°C did not appear to influence cRNA, vRNA and mRNA levels in 293T cell transfected with influenza RNP expression plasmids.

**Table 2:** Effect of a transient heat shock on RNP activity at 42°C.

		mRNA						
(%^a^)/hours		0	1		3		6	
WSN	Control	100	97	±7.8	119	±15.7	133	±16.5
	42°C 15 min	100	103	±8.3	98	±10.8	141	±16.0
HK	Control	100	94	±10.2	115	±21.7	148	±32.7
	42°C 15 min	100	93	±6.4	98	±7.3	127	±24.0

		cRNA						
(%^a^)/hours		0	1		3		6	
WSN	Control	100	112	±6.0	149	±22.4	135	±12.3
	42°C 15 min	100	121	±9.2	115	±18.7	134	±21.3
HK	Control	100	116	±12.8	143	±26.7	151	±10.5
	42°C 15 min	100	97	±8.2	108	±9.2	114	±19.5

		vRNA						
(%^a^)/hours		0	1		3		6	
WSN	Control	100	103	±4.7	118	±14.3	114	±7.0
	42°C 15 min	100	108	±6.4	105	±15.7	109	±15.3
HK	Control	100	105	±16.2	105	±20.8	126	±21.2
	42°C 15 min	100	100	±11.5	106	±15.8	108	±26.0

a The replication (cRNA and vRNA) and transcription (mRNA) activities are indicated as a relative activity (%) ± S.D. from start point (0 hours) (n = 3).

### Analysis of the contribution of the RNA polymerase subunits to thermal stress

It was previously shown that PB2 subunit is required for viral replication at high temperatures [23]. PB2 is also associated with efficient replication of cold adapted influenza virus [24], [25], [26]. More recently, it was suggested that the steady-state level of polymerase - cRNA complex is important for the thermal stability of influenza virus [37]. Thus the PB2 subunit can modulate the stability and/or activity of RNP including RNA polymerase complex under thermal stress. To determine which polymerase subunit(s) are required for stable activity of RNA polymerase on thermal stress in 293T cells, we constituted artificial hybrid polymerases and measured the steady-state levels of mRNA, cRNA and vRNA. Thus the hybrid trimeric complexes consisted of one polymerase subunit from other of the H5N1 polymerase and the other subunits from the WSN polymerase. In all cases the polymerase subunits (PB1, PB2 and PA) were visualized on 7.5% SDS-PAGE by silver-staining, showing that these hybrids could form a functional complex (Results not shown). Furthermore partially purified polymerases were active in an *in vitro* replication assay and in promoter binding (Results not shown).

**Figure 4:**
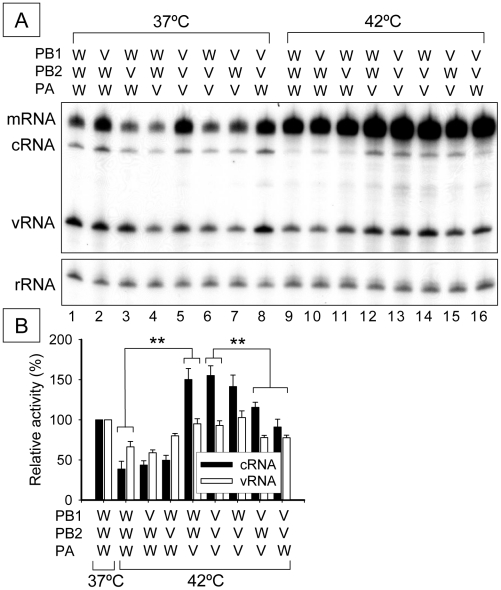
Analysis of the contribution of the RNA polymerase subunit(s) to thermal stress. (A) Representative analyzed polyacrylamide gel (6%) is shown. WSN (W) subunit(s) was replaced with corresponding VN (V) subunit(s). 293T cells expressing the hybrid RNP were incubated at 37°C for 24 hours as pre-incubation. Pre-incubated cells were additionally incubated at 37°C or 42°C for 9 hours. Total RNAs were extracted and analyzed by primer extension assay. Each RNA polymerase subunit is indicated as PB1, PB2 or PA. 5s ribosomal RNA (rRNA) is indicated as an internal control. mRNA, cRNA and vRNA are viral messenger RNA, complementary viral RNA and viral RNA, respectively. (B) Quantitation of cRNA (closed bar) and vRNA (opened bar) and standard deviations of bands in panel A expressed as a percentage of WSN strain at 37°C. ** represents statistical significance at p<0.01 in a Student's t-test (n = 3). Each RNA polymerase subunit is indicated as PB1, PB2 or PA.

At 37°C only the PB1 subunit of VN (V) in a WSN (W) background increased activity (Figure 4A, lane 2). The PB2 and PA subunits were inhibitory (Figure 4A, lanes 3 and 4). In the reciprocal hybrids in a VN background, the PB1 subunit had the most obvious effect causing a decrease in activity (Figure 4A, lane 6) whilst the PB2 subunit had a small inhibitory effect (Figure 4A, lane 7). At 42°C, when WSN PA was substituted with VN PA, in a WSN background, activity was significantly increased (Figure 4A, lanes 9 and 12, and Figure 4B); in contrast the PB1 and PB2 subunits did not significantly affect activity (Figure 4, lanes 10 and 11). In the reciprocal hybrids, activity (notably cRNA) decreased by the substitution of VN PA with WSN PA in a VN background (Figure 4A, lane 13 and 16, and Figure 4B). Taken together, we conclude that the PA subunit of VN appears to be required for optimal activity of RNP when assayed at 42°C.

### The PA subunit of the RNA polymerase can modulate RNP activity under stress

**Figure 5:**
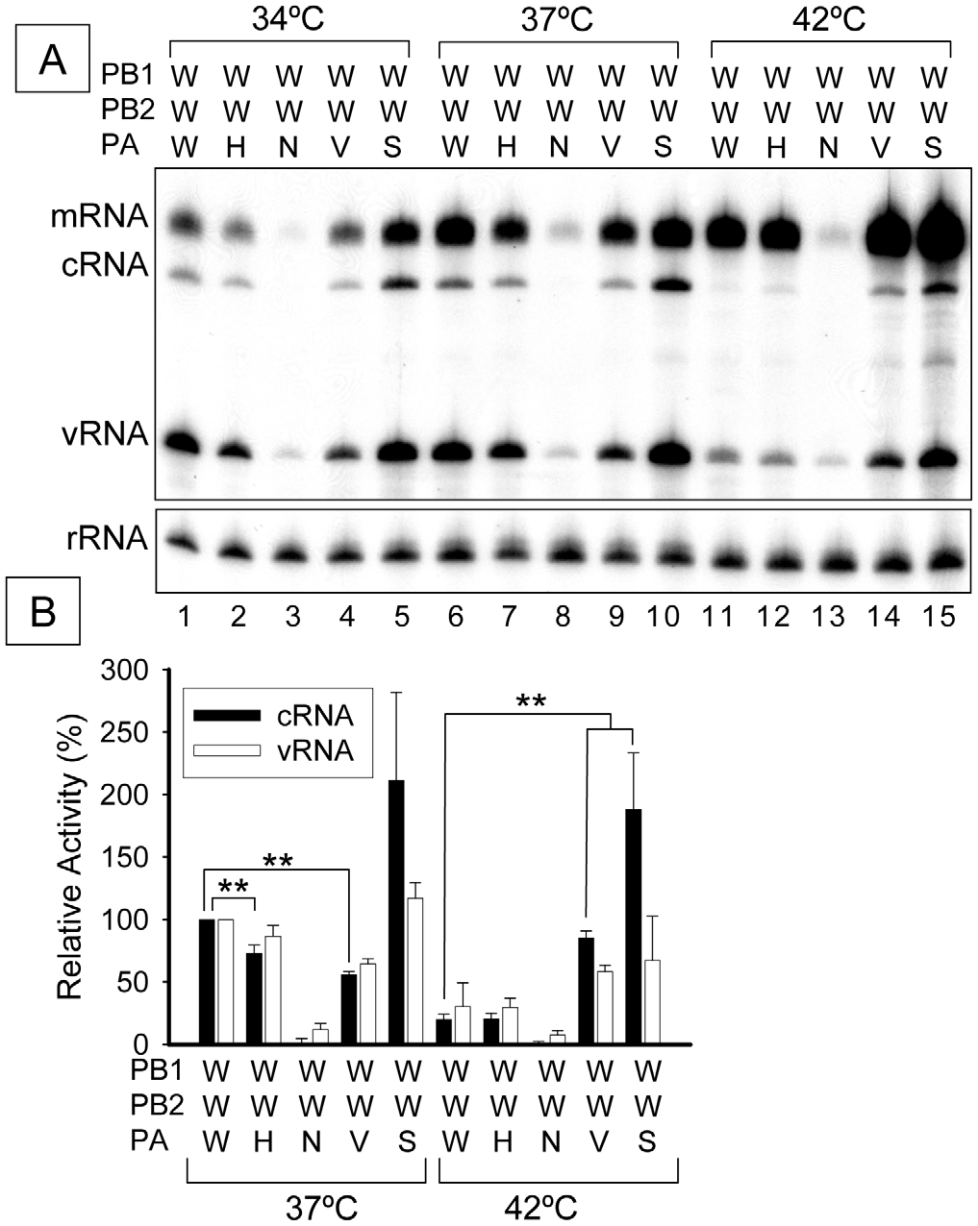
Effect of PA subunit under various thermal stresses. (A) Representative analyzed polyacrylamide gel (6%) is shown (n = 3). WSN PA subunit was replaced with that of each strain. 293T cells expressing influenza RNP were incubated at 37°C for 24 hours as pre-incubation. Pre-incubated cells were additionally incubated at 34°C, 37°C or 42°C for 9 hours. Total RNAs were extracted and analyzed by primer extension assay. A/WSN/33, A/HongKong/156/97, A/NT/60/68, A/Vietnam/1194/2004 and pandemic H1N1 2009 virus are abbreviated as W, H, N, V and S respectively. 5s ribosomal RNA (rRNA) is indicated as an internal control. mRNA, cRNA and vRNA are viral messenger RNA, complementary viral RNA and viral RNA, respectively. (B) Quantitation of cRNA (closed bar) and vRNA (opened bar) and standard deviations of bands in panel A expressed as a percentage of WSN strain at 37°C. ** represents statistical significance at p<0.01 in a Student's t-test (n = 3). Each RNA polymerase subunit is indicated as PB1, PB2 or PA.

Further evidence that the PA subunit can modulate RNP activity was obtained by constructing hybrid polymerases and testing RNP activity in 293T cells under conditions of thermal stress. In Figure 5A and 5B each of 4 different PA subunits – from HK (H), NT (N), VN (V) or SW (S), was in turn substituted for the WSN (W) PA subunit. The NT PA subunit resulted in a nearly total loss of activity (Figure 5A, lanes 3, 8 and 13), This result is consistent with the fact that RNA polymerase activity, and promoter binding, of this WSN/NT PA combination *in vitro* was weak as shown in previous work [31].

Under conditions of thermal stress at 42°C, however, replication (cRNA and vRNA) of WSN (W) hybrids, substituted with PA subunits of either VN (V) or SW (S), the activities of VN and SW PA subunits were particularly pronounced (Figure 5A, lanes 14 and 15, and Figure 5B). Interestingly, the hybrid RNA polymerase containing the SW PA subunit was more active than the parental WSN polymerase at all temperatures tested (Figure 5A, lanes 5, 10 and 15). By contrast, optimal activity of the RNP containing an RNA polymerase substituted with the VN PA subunit was shifted to the higher temperature of 42°C (Figure 5A, lanes 4, 9 and 14, and Figure 5B). We conclude that the PA subunit of the polymerase is involved in modulating the activity of RNP at different temperatures.

### Mutagenesis of the PA subunit and thermal stress

**Figure 6:**
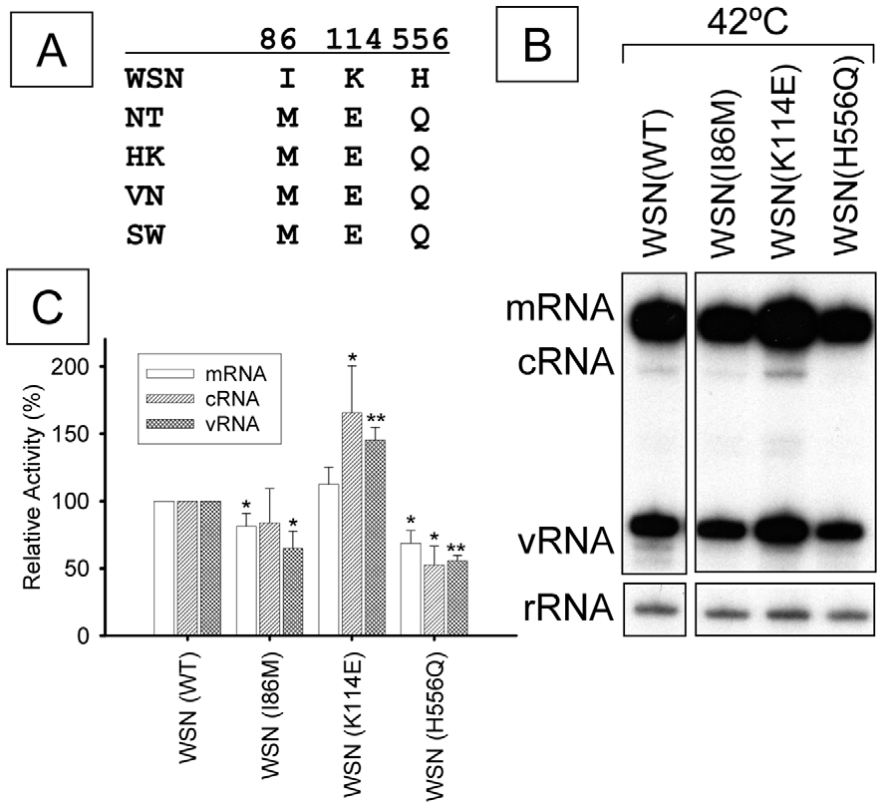
Mutagenesis of the PA subunit and thermal stress. (A) Alignment of PA subunits. Interesting amino acids of WSN PA subunit are indicated. (B) Representative analyzed polyacrylamide gel (6%) is shown (n = 3). WSN PA subunit was mutated at position 86, 114 or 556 as indicated in (A). 293T cells expressing influenza RNP were incubated at 37°C for 24 hours as pre-incubation. Pre-incubated cells were additionally incubated for 9 hours under thermal stress (42°C). Total RNAs were extracted and analyzed by primer extension assay. 5s ribosomal RNA (rRNA) is indicated as an internal control. mRNA, cRNA and vRNA are viral messenger RNA, complementary viral RNA and viral RNA, respectively. (C) The each RNP activity is graphically visualized. Wild type of PA subunit is indicated as WSN (WT). The activity is expressed as a % relative to the WSN wild-type. White, light oblique and dark oblique lined columns show steady-state levels of mRNA, cRNA and vRNA, respectively. * and ** show statistically significant differences from WSN wild-type at p<0.05 and p<0.01 in a Student's t-test.

Since substitution of the PA subunit of different influenza A virus RNA polymerases revealed different properties under conditions of thermal stress, we have extended our analysis of the PA subunit. We aligned the sequences of each PA gene, which highlighted 3 amino acids positions that were identical in NT, HK VN and SW but differed from WSN (Figure 6A). 2 positions, 86 and 114 were in the N-terminal domain of PA that is required for promoter binding and endonuclease activity. The RNP activities derived from each PA mutant identified in this way (I86M, K114 E and H556Q) were introduced into the PA of WSN and were analyzed as before (Figure 6B) and quantitated Figure 6C. Replication (cRNA and vRNA) of the K114E mutant was significantly increased by about 50% by this mutation, whereas both the I86M and H556Q PA mutants decreased activity significantly (Figure 6B and C). Interestingly, the K114E mutant affected only replications (cRNA and vRNA) but not transcription (mRNA), suggesting that replication and transcription were affected independently of one another under conditions of thermal stress. From these findings, we conclude that position 114 of the PA subunit of the RNA polymerase is necessary for the optimal RNP activity for replication under conditions of thermal stress.

## Discussion

Here we test the hypothesis that the optimal temperature of influenza A RNA polymerase differs in different viruses of human origin, and can be modulated by the differing combinations of its polymerase subunits. We analyzed two human isolated H5N1 strains (A/Hong Kong/156/97 [HK] and A/Vietnam/1194/2004 [VN]) and a recently isolated H1N1 swine flu strain (A/Kurume/K0910/2009 [SW]) from humans and compared their properties with two well studied classical, conventional, human isolates of H3N2 origin (A/NT/60/68 [NT]) or H1N1 origin (A/WSN/33 [WSN]). We found, by expressing RNP containing the different polymerases in 293T cells, and analyzing steady state levels of mRNA, vRNA and cRNA at 3 different temperatures (34, 37 and 42°C), that there were clear differences in the levels of RNA expression in the 5 strains tested.

We found that the classical H1N1 WSN polymerase was adapted to the low temperature (34°C), and lost activity rapidly at higher temperatures (42°C). This property may reflect the past history of passage of this strain that was artificially passaged in eggs and then subsequently in the brains of mice. Interestingly, the H5N1 HK strain, isolated in 1997, possessed an optimal temperature in 37°C whereas a closely related H5N1 VN strain isolated only 7 years later preferred 42°C, although we have tested only in human cells not in avian cell. This difference might be caused by the PB2 subunit, because the HK PB2 and VN PB2 differ in the amino acid at position 627, which is glutamic acid (E) in HK and lysine (K) in VN. The SW PB2 contains E at position 627 of PB2 - like the HK strain, yet SW showed a different susceptibility to temperature from WS, HK and VN in that it was active at all 3 temperatures tested. This observation suggests that PB2 627 cannot be the sole determinant of the differences observed here in susceptibility of RNP to temperature in the HK and VN strains. It is known that the 2009 H1N1 virus acquired a second-site mutations in its PB2 subunit at position 590 and 591 to adapt to the human host [47].

In a study of the changes in steady state levels of mRNA, cRNA and vRNA over a 24 hour period at 42°C remarkable differences between the levels of mRNA and replication products (vRNA and cRNA) were observed in the WSN strain, but not in the HK and SW strains tested (Figure 2C). Instead of a synchronized, parallel decrease in mRNA, cRNA and vRNA, as might have been expected because the synthesis of all 3 RNAs are catalyzed by the same RNA polymerase and are interdependent, we observed clear differences in that cRNA levels decreased faster than vRNA in the WSN strain; mRNA levels remained the highest. These observations suggested that vRNA to cRNA synthesis is more sensitive than vRNA to mRNA synthesis in this strain, i.e. replication is more sensitive to heat treatment than transcription. Western-blotting analysis showed that the protein expression levels of RNA polymerase subunits and NP didn't affect both levels of decreased cRNA and increased mRNA at 42°C (Figure 2D). Control experiments, in which we transiently heat shocked RNP, suggested a continuous thermal stress but not a short heat shock is required for the induction of mRNA levels and reduction of both cRNA and vRNA levels, consisting with previous report [37], although it cannot be excluded that heat shock proteins might still be involved.

Why were mRNA levels enhanced by a continuous thermal stress? One of the possible answers may be an altered balance of RNA replication versus transcription in favor of transcription. Similar results have been reported in the closely related A/PR/8/34 (H1N1) strain treated at 41°C [37]. Those authors suggested that polymerase activity was shifted to the transcription because of a reduction of the replication. Interestingly, it is known that the phosphorylated RNA polymerase II large subunit (POLIIo) accumulated under conditions of heat shock [48]. On the other hand, the influenza RNA polymerase bind to the POLIIo at its C-terminal CTD domain for viral transcription [49]. Thus we suggest that the interaction between influenza RNA polymerase and cellular phosphorylated-RNA polymerase II might be promoted by highly temperature thereby causing the increased mRNA levels. Taking our results on thermal stress together, we propose that the mechanisms of the decreased cRNA synthesis and the increased mRNA synthesis are different and independent of one another.

To test which subunit(s) of the RNA polymerase was important for replication and transcription under conditions of thermal stress, we constructed artificial hybrids of RNP and measured their activities. The PA subunit was obviously involved in increased activities in WSN-VN hybrids (Figure 4A and 4B). PB2 did not appear to be involved contrasting with previous studies on cold-adaptation [24], [25], [26]. Perhaps there are differences in the factors influencing stress at low and high temperatures.

We further tested if the PA subunit can modulate the RNA polymerase activity in RNP at different temperatures from 34°C to 42°C. Activity of the RNP clearly depended both on the origin of the PA subunit and the temperature and varied widely from essentially no activity to a much enhanced activity (Figure 5A and 5B). To determine which amino acid of the PA subunit is important for the thermal stability, we focused on positions 86, 114 and 556 - positions that differed between WSN and the other strains studied here (Figure 6A). The K114E mutant was found to promote replicative activity under thermal stress compared with that of wild type (Figure 6B and 6C). The function of this position 114 in the PA subunit has not been analyzed before, but may affect promoter binding [30], [31], [32], or may modulate endonuclease function [17], [28], [29], [30] since both these functions are mediated by the N-terminal domain of PA. The H556Q mutation of WSN PA subunit reduced replication and transcription (Figures 6B and C). This position may interfere or modulate the interaction with hCLE – a host factor thought to regulate the activity of viral RNA polymerase and known to bind nearby [50], [51]. The I86M mutation of WSN PA subunit also reduced replication and transcription (Figure 6B and C), although the reduced level of cRNA was not statistically significant. It has previously been shown that the position 86 locates in the 4th alpha helix which is exposed to the surface of the PA subunit [17], indicating that this region may be associated with the RNA binding.

In summary, we show that particular combination of subunits of influenza RNA polymerase can modulate its thermal sensitivity. In addition, position 114 of the PA subunit is involved in conferring stability to thermal stresses. How does influenza A virus adapt to various hosts that possess different body temperatures? We propose, given the evidence presented in this paper, that in the emergence of new pandemic viruses the subunit(s) of RNA polymerase are reassorted and/or mutated to allow adaptation of the virus to the differing temperature of the new host.
